# Cross-Scale Industrial Manufacturing of Multifunctional Glass Fiber/Epoxy Composite Tubes via a Purposely Modified Filament Winding Production Line

**DOI:** 10.3390/polym16121754

**Published:** 2024-06-20

**Authors:** George Karalis, Lampros Koutsotolis, Angelos Voudouris Itksaras, Thomai Tiriakidi, Nikolaos Tiriakidis, Kosmas Tiriakidis, Alkiviadis S. Paipetis

**Affiliations:** 1 Department of Materials Science and Engineering, University of Ioannina, 45110 Ioannina, Greece; gkaralis@uoi.gr (G.K.); l.koutsotolis@uoi.gr (L.K.); a.voudouris@uoi.gr (A.V.I.); 2 BT Composites S.A., 53100 Florina, Greece; thomai@btcomposites.gr (T.T.); nikos@btcomposites.gr (N.T.); kosmas@btcomposites.gr (K.T.)

**Keywords:** hierarchical fibers, functional coatings, industrial scale-up, filament winding, multifunctional composite structures, crashworthiness

## Abstract

In the present research work is demonstrated a cross-scale manufacturing approach for the production of multifunctional glass fiber reinforced polymer (GFRP) composite tubes with a purposely redesigned filament winding process. Up until now, limited studies have been reported towards the multiscale reinforcement direction of continuous fibers for the manufacturing of hierarchical composites at the industrial level. This study involved the development of two different multi-walled carbon nanotube (MWCNT) aqueous-based inks, which were employed for the modification of commercial glass fiber (GF) reinforcing tows via a bath coating unit in a pilot production line. The obtained multifunctional GFRP tubes presented a variety of characteristics in relation to their final mechanical, hydrothermal aging, electrical, thermal and thermoelectric properties. Results revealed that the two individual systems exhibited pronounced differences both in crushing behavior and durability performance. Interestingly, for lateral compression the MWCNT coatings comprising a polymeric dispersant minorly affected the mechanical response of the produced tubes. The crashworthiness indicators of the multifunctional tubes displayed a slight 5% variation to the respective reference values, combined with a more ductile behavior. Moreover, regarding the bulk electrical and thermal conductivity values, as well as the Seebeck coefficient factor, the corresponding tubes displayed a variance of 233% and 19% and an opposite semi-conducting sign denoting a p- and n-type character, respectively.

## 1. Introduction

Lightweight advanced composites constitute a prominent category of high-demand construction materials with significantly improved specific properties, which combine remarkable mechanical strength and corrosion resistance. Moreover, their facile manufacturing methods and affordable maintenance have led composites to rank first among the state-of-the-art structures capable of a wide range of applications including aerospace, automotive, renewable energy, etc. [1]. Additionally, the requested CO_2_ emissions reduction for transportations and the aspect of sustainability are key objectives for a positive environmental impact that can be reached through their extended of usage [2]. The most prestigious class of polymer-matrix structural composites is that of the continuous carbon fiber reinforced polymers (CFRP). Carbon fibers (CF) are attractive, as they combine low density, high strength and high modulus of elasticity. Furthermore, the intrinsic high electrical conductivity of the graphitized CF filaments microstructure renders CFRP composites electrically conductive in both in-plane and through-thickness directions. Except for their illustrious electrical and thermal conductivity, CF have been also demonstrated as counter electrodes [3], heating elements [4] and p- and n-type thermoelements [5]. A high-volume fraction of the fibers is crucial for attaining high strength and modulus in the composite. Due to the high degree of alignment of the continuous fibers, the continuous fiber volume fraction can be high (i.e., 60%) [6]. Glass-fiber-reinforced polymer (GFRP) composites are distinguished for their exceptional cost-effective strength performance coupled with high stiffness and relatively low humidity absorption. Thus, they are widely preferred for room temperature (RT) and elevated temperature structural and functional applications. Nevertheless, with the constant advancements of technology, higher requirements are being demanded in terms of mechanical strength and electrical-thermal conductivity from various sectors, e.g., aerospace parts, cryogenic tanks, etc. [7]. At the same time, GFRP composites usually present low thermal conductivity, which restricts their implementation in several modern engineering and commercial applications [8]. Moreover, composites science and engineering provide numerous parameters that facilitate tailoring of the final composite properties, such as the volume fractions, fiber orientations, fiber–matrix interface etc. [9]. Consequently, mechanical strength, elastic modulus, thermal expansion coefficient, electrical resistivity and thermal conductivity values are able to be adjusted by design for special applications [10].

Epoxy-resin-impregnated GF and other polymer fibers are less expensive than CF counterparts, but they are not electrically conductive and are therefore not suitable for transfusing additional functionalities (e.g., anti-static charging, electromagnetic shielding, etc.). Multifunctional structural composites constitute an extremely interesting and multidisciplinary field of applied research, introducing extra functionalities like structural integrity monitoring, strain or damage detection, anti-strike protection, electrothermal heating, self-healing or self-repairing, massless energy harvesting and storage [11]. The combined structural and nonstructural features of the next-generation composites are expected to optimize product lifespan and increase product utility with minimum structural degradation. The imparted functionalities may be active, passive or adaptive. In more detail, when a multifunctional structural composite is being exposed to a certain field upon its service life, is able to perform a full cycle of sense–evaluate–react, in response to an external stimulus (i.e., mechanical, electrical, thermal, etc.). Therefore, multifunctionality is the provision of engineering to integrate smart properties at a system level, in order to confer additive value and develop sustainable products for the composite markets [12]. 

For more than a decade, biomimetic nanotechnology concepts have been evolved targeting the incorporation of nanomaterials to enhance multiple advanced structural composites at the macroscale level. Fiber-reinforced polymers (FRP) offer the potential for flexible design via the realization of novel fabrication approaches, which empower multiscale reinforcements. Carbon nanotubes (CNT) have been suggested as ideal candidates for further enhancing FRP composites, due to their outstanding physical properties (i.e., high aspect ratio, Young modulus, tensile strength, electrical and thermal conductivity) [13]. Thus, significant improvements in specific properties can be achieved, i.e., enriched fracture toughness, interfacial adhesion, and electrical and thermal characteristics [14,15]. Representative research efforts are associated with the dispersion of carbon nano allotropes as fillers into the polymer matrix [16], known as the hybrid approach, aiming to improve matrix properties. Indicatively, Thomas and colleagues reported that the addition of surfactant-modified CNT into the epoxy caused an increase in the mechanical and thermal properties on account of an improvement mechanism attributed to the uniform dispersion of the CNT in the epoxy matrix minimizing the free volume between the polymer chains and the respective mobility. This free volume restriction seems leading to a stronger interfacial bonding and more efficient load transfer capability between the CNT filler and epoxy matrix up to a certain filler concentration level [17]. Considerable attention has also been focused on wet-chemical deposition techniques as CNT coatings onto the fibrous reinforcing phase [18], known as the hierarchical approach, where the main objective is to directly improve the interface with the formation of a tougher and more functional one.

A few studies regarding hierarchical reinforcing concepts have been conducted by several research groups, e.g., [19]. Qin et al. developed a scalable electrochemical anodization-based manufacturing process for the in situ growth of CNT onto continuous CF, thus improving both the tensile strength of the CF–CNT reinforcement, as well its wettability. This led to a 95.43% increase of the interfacial shear strength (IFSS) [20]. Another relevant study has dealt with the electrophoretic deposition (EPD) method used to deposit polyethyleneimine (PEI)-functionalized MWCNT coatings onto individual GF. Optimizing the parameters of the EPD method resulted in a composite with 58% increased IFSS. Moreover, the introduced electrical sensitivity enabled damage initiation detection [8]. Pegoretti and coworkers reported a novel nanomaterial deposition technique involving the triboelectrification of GFs to positively charge their surfaces in order to facilitate negatively charged graphene oxide (GO), dispersed in an ink. The GO coating was then chemically treated to reduce graphene oxide (rGO). The GF surface modification resulted in a 45% increase of the fiber–matrix adhesion and a minor improvement in the electrical conductivity [21].

Hierarchical reinforcing approaches may be combined with high-productivity, low-cost industrial manufacturing techniques, such as filament winding, to produce composite materials on a mass scale. Filament-wound composite tubes have been widely considered due to their outstanding energy absorption capacity and crashworthiness characteristics. Gupta and Abbas investigated the lateral crushing behavior of GFRP tubes as a function of their geometrical features [22], while Li et al. studied the effect of the geometry on CFRP, GFRP and aluminum (Al) tubes [23]. The crushing response and the failure modes of filament-wound CFRP, CFRP/Al and GFRP/CFRP/Al hybrid tubes under axial compression is also reported in the literature [24,25]. The scope of the present research work was to investigate the potential for cross-scale production of hierarchical GFRP tubes relying on the well-established and purposely modified industrial filament winding technique. Scientifically, the main innovation point focuses on the realization of multiscale reinforcements towards the introduction of manufacturing multifunctional composites at an industrial scale. Hence, two aqueous-based MWCNT inks were prepared, differentiated in their dispersant selection and concentration in order to be employed for the production of reinforcing GFs coated with MWCNT-based nanostructures via a low-cost continuous bath coating stage. Subsequently, the manufactured multifunctional GFRP tubes were examined with respect to their attained mechanical, hydrothermal aging, electrical, thermal and thermoelectric properties.

## 2. Materials and Methods

### 2.1. Materials

The E-GF 1062 Multi-End Roving (Ōtsu, Japan) by NEG was employed in this work with 2300–2400 MPa tensile strength and 72 GPa tensile modulus, having been purposely produced for utilization with epoxy-based resin matrices in high-demand applications, where mechanical strength and corrosion resistance are the main requirements. The mean filament diameter is ca. 13.3 μm. The EPIKOTE Resin 828LVEL (100.0 parts per weight), EPIKURE Curing Agent 866 (83.0 parts per weight) and EPIKURE Catalyst 101 (1.5 parts per weight) low viscosity epoxy system by Hexion (Columbus, OH, USA) were selected. The curing and post-curing procedure consist of heating cycles at 90 °C for 90 min and at 130 °C for 180 min, respectively. As stated within the manufacturer’s technical datasheet, the glass transition temperature (T_g_) of the thermoset matrix system after the proposed curing cycles is in the range of 152–156 °C. MWCNT NC7000™ in the form of powder (carbon content: 90 wt%), with an average diameter of 9.5 nm and an average length of 1.5 μm, was provided by NANOCYL. Sodium dodecylbenzene sulfonate (Mw = 348.48 g/mol), abbreviated as SDBS, and polyvinylpyrrolidone (M_w_ = 10,000 g/mol), abbreviated as PVP, were received from Sigma–Aldrich (St. Louis, MI, USA). Distilled (DI) water has been used throughout the dispersion processes. Nitric acid (65%) from Lach-Ner Ltd. (Neratovice, Czech Republic). chemicals was utilized for fiber volume fraction determinations. Silver (Ag) conductive adhesive epoxy-based paste by RS Pro was used for the creation of proper ohmic contacts for the electrical and thermal measurements.

### 2.2. Preparation of the Aqueous MWCNT-Based Inks 

MWCNT powder was dispersed in DI water using the tip-ultrasonic probe processor UP400S by Hielscher (Teltow, Germany) for 30 min at 10-Watt amplitude and overnight mechanical gentle stirring at ambient conditions. For the first case, a DI water dispersion of 3 mg/mL MWCNT containing 6 mg/mL SDBS anionic surfactant was employed to produce a stable ink, denoted henceforth as MWCNT:SDBS. Proportionally, for the second case, a DI water dispersion consisting of 3 mg/mL MWCNT and 9 mg/mL PVP as stabilizing dispersant were combined for the preparation of a slightly thixotropic stable ink, denoted as MWCNT:PVP. The amorphous, water-soluble and hygroscopic PVP presents a T_g_ in the range of 110–180 °C depending on the molecular weight [26].

### 2.3. Cross-Scale Manufacturing of the Multifunctional GFRP Tubes

**Figure 1:**
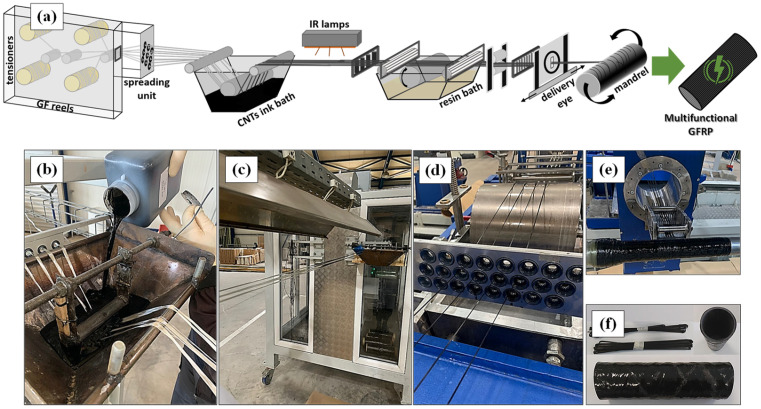
(**a**) Schematic representation of the purposely modified filament winding production line and various snapshots of the redesigned filament winding steps for the cross-scale manufacturing of multifunctional GFRP tubes: (**b**) MWCNT-based ink bath for GF tows coating process after the electronic tensioner, (**c**) high-temperature drying system with IR lamps for the rapid aqueous solvent evaporation, (**d**) resin bath impregnation of the coated GF-MWCNT tows and guidance to the ceramic ills for protection and further alignment, (**e**) winding process and (**f**) final coated GF-MWCNT tows and multifunctional GFRP tubes.

GFRP multifunctional composite tubes were manufactured at the B&T Composites S.A. company facilities, located in Florina, Greece, using a purposely transformed filament winding pilot production line (see Figure 1). Two different series of multifunctional GFRP tube systems were manufactured. For the first one, the MWCNT:SDBS ink was employed, reffered to as system 1, and for other the MWCNT:PVP ink, labeled as system 2. From a technical perspective, the speed of the production pilot line during the continuous manufacturing process was 0.5 m/min. Initially, gentle mechanical stirring for one hour was performed for each developed ink before the ink bath feed in order to reduce the dynamic viscosity during the coating procedure step of the under-tension GF tows (Figure 1b). Thereafter, the tensioned and fresh-coated GF tows passed under a 7 m array of IR lamps fixed at 380 °C for the completion of the drying step, as presented in Figure 1c. Because the fibers become less flexible for winding due to the coating and drying stages, a comb of vertical ceramic cylinders was interpolated between the IR lamps and the resin bath in order to reduce their stiffness, thus resulting in better impregnation. The resin bath was appropriately modified with a metal structure comprising additional winding rollers that further improve the impregnation of the coated fibers. Afterwards, the resin bath impregnation step occurred for the coated GF-MWCNT tows, which were guided through a stencil of ceramic ills for protection and further alignment (Figure 1d). Then, upon the filament winding technique, a robotic arm with a delivery eye (Figure 1e) applied the resin-impregnated coated GF tows onto a spinning metal mandrel at a specific inclination angle for each layer following a predefined layup. The GF tows’ winding angles were ±55° and ±80°, and the winding layers, i.e., the number of turns of the GF tows around the mandrel mold, were four. More precisely, the sequence of the four layers was +80°, −80°, +55° and −55°, a sequence considered optimal in filament-wound composite tubes [25]. Finally, the composite tube was subjected to thermal curing and post-curing cycles at 90 °C for 90 min and at 130 °C for 180 min, respectively. Simultaneously, the structure was continuously rotated along its longitudinal axis to evenly ensure the appropriate resin content. Figure 1f depicts the coated GF tows and the produced multifunctional tubes. The corresponding conventional GFRP composite tubes, excluding the bath coating step, were also manufactured for comparison. More details regarding the geometrical parameters of the produced tubes are included in the Supporting Information (Figure S1 and Table S1).

### 2.4. Characterization Methods

#### 2.4.1. Rheology

The dynamic viscosity of the MWCNT-based aqueous inks used for the bath coating process was measured with an NDJ-9S digital rotary viscometer (Jinan, China).

#### 2.4.2. Analytics

Spectroscopy measurements for the coated GFs were carried out with the Labram HR-Horiba scientific micro-Raman system (Kyoto, Japan). The 514.5 nm line of an Ar+ ion laser operating at a laser power of 1.5 mW at the focal plane was employed for the Raman excitation. An optical microscope equipped with a 100× long working distance objective served both for delivering the excitation light and collecting the back-scattering Raman activity. All Raman spectra were collected in the range of 100–3500 cm^−1^.

Thermogravimetric analysis (TGA) mass loss tests for coated GF tows were performed with the STA 449C analyzer Netzsch Gerätebau GmbH (Selb, Germany). The experimental procedure included a dynamic step from 25 to 1000 °C with a heating rate of 10 °C/min under O_2_ flow.

Fiber volume fraction of the different tubular systems was calculated based on ASTM D3171-15 [27]. To do so, samples were extracted from the tubes and weighted. Subsequently, they were immersed in nitric acid for the matrix to dissolve. Following the full dissolution of the matrix, the remaining fibers were thoroughly cleaned with DI water until the acid was completely removed. After drying, the weight of the fibers was measured.

#### 2.4.3. Morphology

Morphology inspection of the bare and coated GFs was accomplished using the Phenom XL Desktop Field Emission Scanning Electron Microscopy (FESEM) instrument from Thermo Scientific (Waltham, MA, USA) at an operating voltage of 10 kV.

Micro-computed tomography (μCT) scans were conducted with Bruker SkyScan1275 (Billerica, MA, USA). X-ray imaging of specimens was conducted prior to and after mechanical testing. An accelerating voltage of 90 kV, a current of 111 μA and an exposure time of 360 ms were chosen as scanning parameters. The NRecon (Version 2.0) reconstruction software, implementing the Feldkamp algorithm, was utilized to obtain the 3D models from tomography projection images. To visualize the scanned specimens after reconstruction in three and two dimensions, the CTvox and the DataViewer imaging software were used, respectively. The CTAn application was employed to calculate the porosity of the undamaged tubes.

#### 2.4.4. Durability

Compression tests were performed using a Jinan WDW 100 (Jinan, China) universal testing machine with a loading capacity of 100 kN. A constant crosshead speed of 2 mm/min was used for axial compression and a speed of 4 mm/min for lateral compression. For each tubular system and each loading direction, 5 specimens were extracted and tested, adding up to a total of 30 specimens tested under ambient conditions (23 ± 3 °C, 50 ± 10% RH). The crushing behavior of the specimens was videotaped and snapshots of consecutive deformation stages were captured to study the various failure modes.

In order to evaluate the durability of the tubes against environmental exposure, tubes of the three systems were subjected to hydrothermal aging. Conditioning was performed in a BINDER KBF P 720 constant-climate chamber. The temperature was set at 70 °C and the RH at 85% according to the ASTM D5229/D5229M−14 standard [28], and three tubular specimens of each system were exposed to hydrothermal aging until moisture saturation content was achieved (see Supporting Information file for more details).

#### 2.4.5. Conductivity

The electrical conductivity of MWCNT:SDBS and MWCNT:PVP in the form of buckypaper films (diameter: 47 mm) with a thickness of ca. 80 μm, resulting from a vacuum filtration process, was exported by measuring the room temperature sheet resistance (Rs) using the four-point probe commercial system by Ossila Ltd. (Sheffield, UK).

Electrical DC measurements were conducted with a standard two-probe method using the Agilent 34401A 6½ digital multimeter (Santa Clara, CA, USA). The multifunctional GFRP tubular specimens were measured at 55 mm (in-plane direction) and at ca. 1.6 mm (through-thickness volume) electrode–electrode distance. Ag paste was applied on the measurement points, which were prior-prepared via slight surficial etching with a Dremel rotary tool for the establishment of ohmic contacts.

Bulk thermal conductivity measurements were carried out with a TCi thermal conductivity analyzer by C-THERM. The GFRP samples were cut at 20 × 20 mm^2^ and Ag paste was applied on the top and bottom side for the formation of thermal contacts.

Thermoelectric in-plane (interelectrode distance: 55 mm) and through-thickness (interelectrode distance: ca. 1.6 mm) DC voltage output of the multifunctional GFRP tubes was acquired using the Agilent 34401A 6½ digital multimeter. For the creation of temperature difference, a commercial metallic-alloy-based electrothermal heating element was surface-mounted on the hot side of every specimen and the thermal activation was achieved by the Xantrex XPH 18−10 DC power supply unit. The cold side of the tube was left under natural cooling at ambient conditions. The temperature of each side was monitored via a Fluke 289 True RMS multimeter with a surface-mounted K-type thermocouple.

## 3. Results and Discussion

For the realization of multifunctional GFRP composite tubes produced by cross-scale industrial manufacturing, the effective exploitation of MWCNT unique properties via a wet chemical deposition approach is vitally important. In more detail, the addition of an anionic surfactant (i.e., SDBS) in DI water due to π-stacking bonding confers a negatively charged character to the generated micelles, resulting in the promotion of de-aggregated MWCNT dispersion [29]. On the other hand, the incorporation of a polymer (i.e., PVP) in aqueous medium owing to polymer wrapping bonding phenomena permits the actualization of highly dispersed and slightly thixotropic ink, mainly due to polymer swelling combined with electrostatic interactions [30]. The dynamic viscosity values of the MWCNT:SDBS and MWCNT:PVP inks utilized for the bath coating deposition process were 240.2 and 384.6 mPa.s, measured at RT. The corresponding electrical conductivity values of the derived bulk films were 1.64 × 10^3^ S/m and 4.42 × 10^3^ S/m, respectively. On the other hand, the positively charged free silanol groups from the GF surfaces interact with the ambient oxygen molecules, causing non-covalent modifications upon deposition [31]. The produced coated GF-MWCNT tows were examined morphologically, spectroscopically and thermally in order to assess their physicochemical properties. Accordingly, the resulting system 1 and system 2, compared with a conventional reference GFRP tube, were tested regarding their mechanical and fracture behavior. In terms of the imparted functionalities, due to the existence of conductive coatings, electric, thermal and thermoelectric characterization was performed for both system 1 and system 2 multifunctional GFRP tubes.

### 3.1. SEM Inspection for the Coated GF-MWCNT Tows 

**Figure 2:**
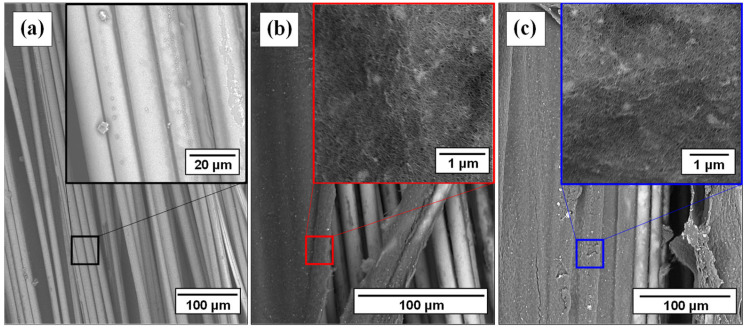
FESEM captures at different magnifications for the (**a**) bare GF tow, (**b**) coated GF-MWCNT:SDBS tow and (**c**) coated GF-MWCNT:PVP tow.

As was noticed by SEM imaging, on both coated MWCNT:SDBS (Figure 2b) and MWCNT:PVP (Figure 2c) microscale GF surfaces, a continuous and dense MWCNT nanostructured network was revealed. Defined agglomerates are barely visible, mainly due to the possible non-reacted excess of the surface-active agent and the polymeric dispersant substance [32] for each case, respectively. The resulting multiple MWCNT charge transport pathways are able to effectively regulate the intrinsic dielectric character of the bare GF tows (Figure 2a) into satisfactorily conductive reinforcing phases.

### 3.2. Spectroscopic and Thermogravimetric Analysis of the Coated GF-MWCNT Tows 

**Figure 3:**
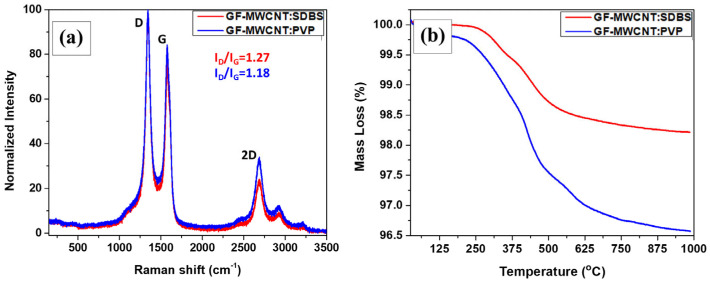
(**a**) Raman spectra and (**b**) TGA measurements for the coated GF-MWCNT:SDBS and coated GF-MWCNT:PVP tows.

Raman spectroscopy was used to evaluate the quality of the graphitic nanostructures after processing, as well as at atomic level the possible interactions between the MWCNT and the respective employed additive for each case. Figure 3a shows the value, normalized to 100, with respect to the intensity of the most prominent peak in each Raman spectrum of the coated GF-MWCNT:SDBS and coated GF-MWCNT:PVP tows, respectively. The radial breathing mode (RBM) peaks at around 100–300 cm^−1^ were undetected, identifying the MWCNT type, as opposed to the single-wall carbon nanotube (SWCNT) type, where RBM features are expected [33]. Namely, the D-, G- and 2D-band peaks were located at ca. 1342 cm^−1^, 1575 cm^−1^ and 2688 cm^−1^, and these signals are typical for MWCNT. The D-band (D represents defect) at the 1300–1400 cm^−1^ region results from a disorder-induced double-resonant procedure attributed to the breakdown of the common wave vector selection rule (i.e., A_1g_ symmetry) [34]. The G-band (G represents graphitic) at 1500–1600 cm^−1^ and the 2D-band at 2300–3000 cm^−1^ are assigned to overtones and combinations of the D- and G-bands. The graphitization and crystallinity degree relating to sp^3^/sp^2^ carbon atoms of MWCNT have been associated several times with the intensity ratio (I_D_/I_G_) of the corresponding G- and D-bands [35]. In particular, the D- to G-band intensity ratio decreases from 1.27 for the coated GF-MWCNT:SDBS tows to 1.18 for the coated GF-MWCNT:PVP tows. This can be explained by a greater amount of induced defects and existing sp^3^ hybridized carbon atoms upon non-covalent functionalization of the MWCNT frameworks, which may further impact the MWCNT charge carrier transport properties affecting the measured electrical conductivity values, as reported previously [36]. It should be highlighted that no evidence of specific vibrational peaks of the utilized surfactant and the corresponding polymer substance were observed, on account of the highly intense MWCNT characteristic signals.

Thermal stability tests for the produced GF-MWCNT tows were performed. As shown in Figure 3b, evaporation of residual moisture was observed to be increased in GF-MWCNT:SDBS due to the increased percentage of carboxy moieties. E-GFs lost around 0.7% of their mass due to the burning of the silane-type sizing [37]. Then, the mass of the E-GFs remained almost unaltered up to 1000 °C. The thermal degradation of the GF-MWCNT:SDBS exhibited the typical decomposition trend of pristine MWCNTs, which are inherently enriched with adsorbed oxygen moieties able to enhance the injection of holes as main charge carriers [38]. Another ca. 1% mass loss up to 375 °C and 530 °C could be attributed to oxygen-containing functional groups like hydroxy or carboxyl and ester moieties [39]. The initial mass loss of SDBS molecules is caused by the combustion within the O_2_ environment in the range of 330–450 °C and the gradual burning occurring at ~800 °C [40]. Concurrently, a ca. 0.8% mass loss of amorphous carbon up to 760 °C may have occurred because of the MWCNT combustion. Lastly, a further ~0.2% mass loss could be attributed to some possible catalyst particles remaining, originating from the MWCNT synthetic process [41]. A slightly different behavior was mentioned for GF-MWCNT:PVP. Under O_2_ flow, a two-stage degradation procedure of PVP was visible. The mass loss began at ~220 °C up to 380 °C, while the oxidation of PVP molecules led to carbon dioxide and pyrrolidone around 300–400 °C [42], and the decomposition of PVP caused by the collapse of the side groups. 

### 3.3. Crashworthiness and Energy Absorption of the Multifunctional GFRP Tubes 

**Figure 4:**
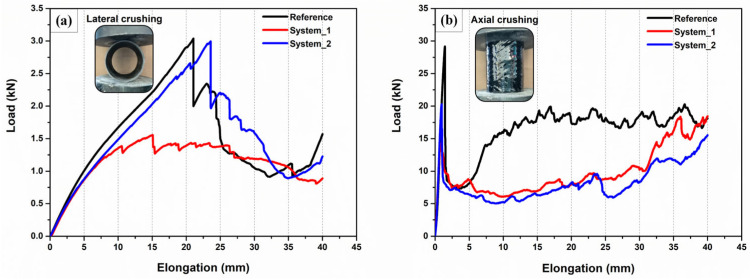
Representative load-displacement curves for (**a**) lateral and (**b**) axial crushing.

Figure 4 illustrates representative load-displacement curves under both lateral and axial crushing; the corresponding crashworthiness indicators are summarized in Table 1 (for more information, see Tables S2 and S3). 

As can be seen, the modification of the GFs with MWCNTs affects the mechanical response of the tubes. At lateral compression, system 1 displays a clear deterioration in mechanical properties. Both the peak crushing force (PCF) and the energy absorption (EA) are lower than the respective reference values, with the PCF being halved while the EA is reduced by 17.5%. The force-displacement curve does not follow the typical pattern, as multiple successive failures of low intensity can be distinguished instead of the two large ones corresponding to the formation of the two fracture lines. Additionally, the first significant failure, chosen as the PCF, does not always coincide with the maximum compressive load. The degradation of the mechanical behavior is due to the SDBS residuals, which hinder to some extent the resin impregnation of the fibers, leading to structural elements of lower interfacial shear strength [29]. On the other hand, the use of an ink comprising a polymeric dispersant only minorly affects the mechanical response of the system 2 tubes. The crashworthiness indicators show a variation of about 5% from the respective reference values, which may be considered negligible, while the load-displacement curve exhibits no qualitative differences with the reference. Moreover, the system 2 tubes are slightly more ductile, since the first significant failure occurs at larger displacement. Under axial compression, both tubular systems comprising modified GFs are mechanically degraded. The PCF of system 1 is almost halved in comparison with the reference, while system 2 exhibits an approximately 33% reduction. The absorbed energy is almost the same for both modified systems and about 30% lower than the respective reference values, a reduction owing to the different failure modes exhibited by the modified tubes. The load-deformation curve of the reference and the modified tubes possess a qualitative difference. In the former, after the maximum load, there is a distinctive rerise in the loading capacity, as the remaining healthy material is stiff enough to withstand this loading level. Moreover, later, during the second half of the progressive collapse stage, large load fluctuations appear due to local buckling phenomena.

### 3.4. Fracture Investigations for the Multifunctional GFRP Tubes 

**Figure 5:**
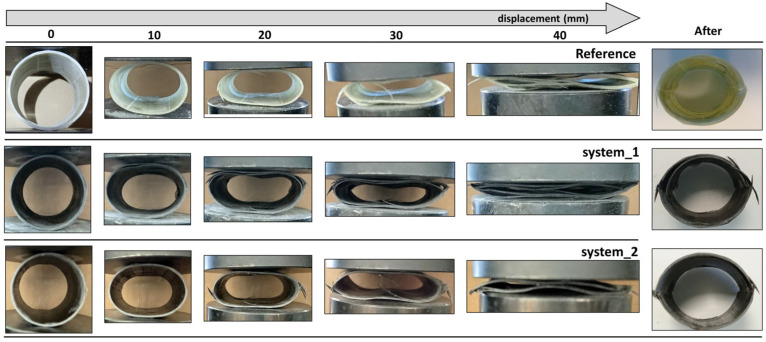
Crushing history under lateral compression.

Figure 5 shows successive snapshots of the lateral crushing process at displacements of 0, 10, 20, 30, 40 mm and after testing. The system 2 tubes undergo a similar failure process with the reference tubes having more brittle crushing behavior and less delamination. The deformation history involves the flattening of the walls in contact with the testing machine plates. The first failure occurs at those areas due to high stress concentration [23,43] and is a combination of local delamination and fiber fracturing. Having the contacting areas as an onset point, delamination propagates circumferentially. Given the D/t ratio, sudden catastrophic failure occurs along the entire length of the tube [22], with the development of two longitudinal fracture lines located at 180° phase angle. The plastic hinges involve fiber breakage and matrix cracking, but delamination is also present at those areas. Furthermore, cracks initiate from the fracture lines and propagate along the fibers in a lengthwise direction. Apart from those failures, the rest of material still remains in the elastic region, which becomes evident by the significant deformation recovery after the end of the test. Contrariwise, the system 1 tubes exhibit dissimilar failure mechanisms, as the collapse is progressive. The main failure mode here is delamination, which is much more extensive than in the other two tubular systems. Delamination appears on the upper and lower side of the tube and propagates circumferentially within its walls. Delamination releases the stored energy and relieves the local stress, preventing the fibers from fracturing. As a result, no catastrophic failure is induced. The fracture lines appear only during the last stages of the test, while the cross-section is not completely fractured, but characterized by severe delamination. When the load is removed, the tube recovers a large part of its deformation. At this point, the existence of detached unbroken fibers protruding from the tube is observed.

**Figure 6:**
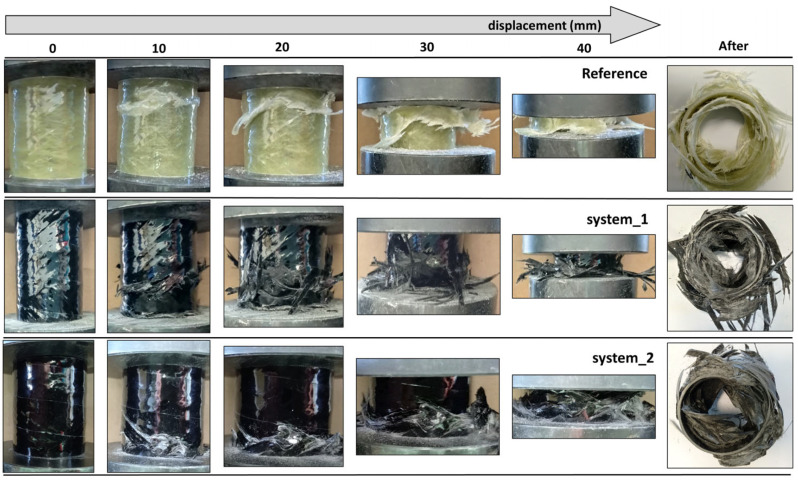
Crushing history under axial compression.

The main energy absorption mechanism in axial crushing is progressive collapse, where the material deforms plastically in a controlled and stable manner, absent of sudden catastrophic failures. Energy absorption involves a combination of micro-cracking, bending or buckling, delamination and friction [44]. The deformation history under axial crushing is illustrated in Figure 6. In general, six failure modes (Mode I–VI) can be identified, each of which corresponds to different energy absorption and damage mechanisms [24]. Their development depends on the D/t ratio and the stacking sequence. In this study, mainly Mode II and IV appeared. Mode II is distinguished by progressive brittle fracturing owing to transverse shearing, bending of the walls and delamination due to local stress concentration, and leads to significant energy amounts being absorbed. Mode IV is characterized by local buckling of the walls that, however, does not instigate global instability to the tube.

The reference tubes exhibited Mode II failure, while their behavior is the most brittle. At the maximum load, due to high shear stress concentration, sudden catastrophic failure occurs in the form of a horizontal fracture line at the weakest cross-section. The progressive crushing of the tube initiates from this line and extends along the longitudinal direction, with the cracks propagating parallel to the fibers. Delamination is also present throughout the fracture zone. Brittle fracturing creates fragments that fall off from the tube. The failure mode of system 1 tubes is a combination of Mode II and IV. More precisely, the collapse includes both brittle fracturing and local buckling of the tube. As the tube is compressed, considerable buckling deformation develops around a horizontal zone, eventually leading to the formation of a fracture line. The progressive collapse begins at this area and proliferates in the axial direction. Despite the fact that after this event the crushing is dominated by progressive collapse, buckling phenomena reappear at some point. This may result in the formation of a new fracture line, in which case the collapse progresses around both. The reoccurring of local buckling is also signified by the large force fluctuations in the load-displacement curves of Figure 4. The crushing features of the system 2 tubes are similar to those of system 1, since here the failure process is also a combination of Mode II and IV. However, the collapse is characterized by more pronounced deformation. In particular, buckling of the walls emerges in various locations. The fracture line develops in the area with the most intense deformations. A second, lesser fracture line may appear in another cross section. The tube will then collapse around both failure zones, with the first one remaining dominant. Fiber fracturing and matrix cracking cause the detachment of material. 

**Figure 7:**
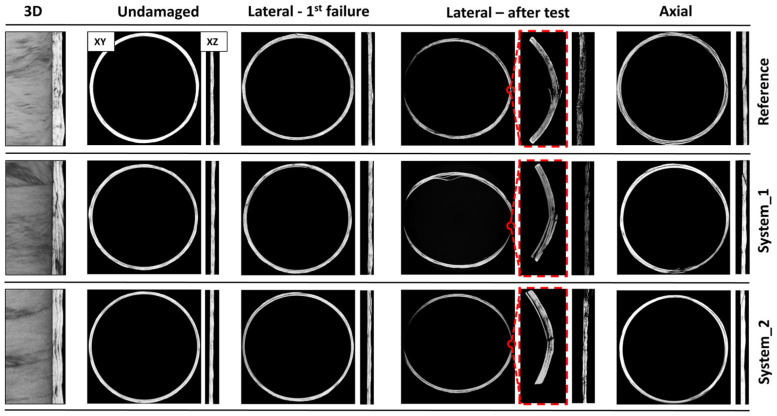
3D and cross-sectional images of the horizontal (XY) and the vertical (XZ) plane. Tubes are shown in the undamaged state, after the first failure under lateral compression, after test and at the end of the test under axial compression.

X-ray imaging reveals important attributes of the failure mechanisms and validates the results of the preceding mechanical evaluation. Figure 7, on the left, shows three-dimensional reconstructed images of the undamaged tubes, revealing their internal structure.

The varying brightness of the captured μCT images owes to the difference of the X-ray attenuations and reflects the different densities of the composite material constituents [45]. Therefore, the high-density fiber regions are shown in light grey, while voids are highlighted in black. Interlaminar voids are large, elongated discontinuities, localized in the areas between layers. Those discontinuities are larger and much more pronounced in the tubes comprising modified GFs. Smaller, round voids, noted as resin voids, can only be distinguished in the reference tube and are scattered throughout the interlaminar area. Cross-sectional images of the horizontal (XY) and the vertical (XZ) plane of the reconstructed tubes are also depicted in Figure 7. The interlaminar areas of the reference tube are not so easily identified, whereas in the modified tubes they are clearly discerned throughout the material, revealing the qualitative difference in the achieved consolidation. The aforementioned difference is also reflected by the achieved volume fractions for each case. Images labelled as first failure present the internal structural state of the tubes after the first major damage occurrence at PCF. The regions with damage present at this stage are the ones in contact with the testing machine platens. Damage is a combination of localized fiber fracturing and delamination initiating from the contact points and extending within the walls. However, fracturing is limited only to the outer part of the wall; thus, it does not lead to the formation of a fracture line. The two fracture lines are formed at a later stage and are visible in the after-test images. The reference and the system 2 tubes that presented the same failure mechanisms displayed a similar stage of damage. The plastic hinges, shown in the enlarged images, are well-defined and separate the tube into two sections. Delamination at those areas is also present. The unmodified tubes behaved more brittlely, meaning they exhibited extensive fiber fracturing and less delamination. In system 1, delamination is widespread throughout the entire wall of the tube. Conversely, fiber breakage is rather confined. The images taken after the axial crushing reveal the nature of the fracture line. Multiple fiber breakage as well as delamination are clearly identified. Fiber fracturing is more intense in the reference tube that has the most brittle crushing behavior. Broken fibers are clustered owing to stress concentration at those areas. The failure zone of the system 1 tube is, on the other hand, characterized by heavy delamination. The damage type of the system 2 tubes lies between the two other categories. The vertical cross-sections of all three examined tubular systems validate the Mode II failure that occurs. The fracturing of this failure mode is attributed to transverse shearing and leads to an approximately 45° splitting of the tube (XZ plane). The total porosity for the reference, system 1 and system 2 tubes was calculated by image processing as 8.47%, 20.39% and 10.67%, respectively. The considerable amount of the void content is a result of the weaker compaction achieved by the filament winding manufacturing process [46]. The substantial increase in the porosity of the system 1 tubes owes to the employment of the SDBS dispersant that results in poorer impregnation of the coated GFs. Meanwhile, system 2 ink that utilizes PVP leads to an only 25.97% increased porosity. 

### 3.5. Hydrothermal Durability Assessment for the Multifunctional GFRP Tubes 

**Figure 8:**
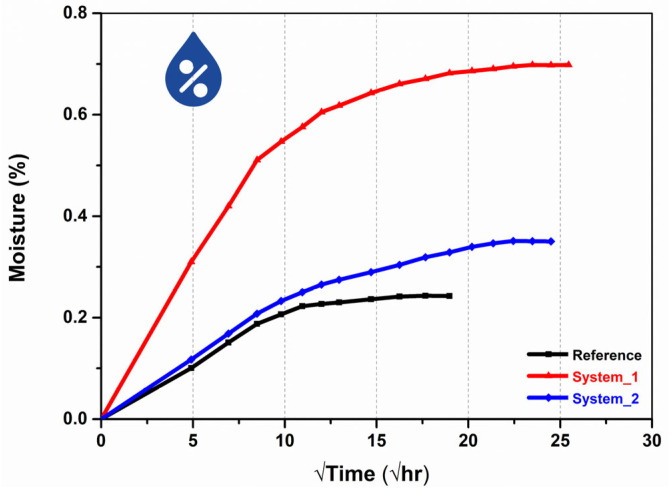
Moisture absorption as a function of the square root of the exposure time.

The moisture absorption curves of the three examined tubular systems are depicted in Figure 8. During the initial absorption stages, the curves are linear and the diffusion process is described by Fick’s second law. The diffusion coefficient (D_z_) can therefore be calculated by the slope of the absorption curves through the equation: D_z_ = slope^2^·(π·t^2^)/16W_∞_ [47], where W_∞_ is the moisture content at the saturation stage and t is the thickness of the tube. The modifications of GFs with MWCNT-based coatings alters the diffusion properties of the composites. In more detail, the equilibrium moisture content of the reference specimens is 0.24%, while saturation is achieved after 15 days. On the other hand, the durability of the modified tubes against hydrothermal exposure is reduced, as implied by the increased moisture content. The system 1 specimens absorb 0.7% water until saturation, while the moisture equilibrium content of system 2 tubes is 0.35%. Saturation stage is reached after 27 and 25 days, respectively. Therefore, the water uptake for system 1 is about three times higher than the reference system, whereas for system 2 there is a 46% increase. Moisture absorption in epoxy-based composites can result in matrix swelling and reduce the fiber–matrix interfacial strength [48]. The diffusion coefficients for the reference, system 1 and system 2 tubes were calculated as 0.010 × 10^−3^ mm^2^/h, 0.028 × 10^−3^ mm^2^/h and 0.008 × 10^−3^ mm^2^/h, respectively. The GF modification has a dual effect on the water uptake at the saturation stage. The inclusion of MWCNT may create a tortuous path for diffusion with longer travelling distance, thus slowing down the time required to reach moisture equilibrium [47]. Conversely, MWCNT-based coatings may increase the interfacial region within the composite, leading to higher water uptake [49], as is the case for system 2. The large increase in moisture absorption that system 1 displayed in comparison to both the reference and system 2 specimens also relies on the hydrophilic SDBS residuals, which hinder GF wetting and consolidation. System 1 presented the lowest mechanical strength in lateral and in axial compression, as well as the highest porosity. Therefore, the hydrothermal aging results are in strong agreement with the mechanical evaluation, the μCT porosity calculation and the calculated fiber volume fractions. 

### 3.6. Electric and Thermal Properties of the Multifunctional GFRP Tubes 

**Figure 9:**
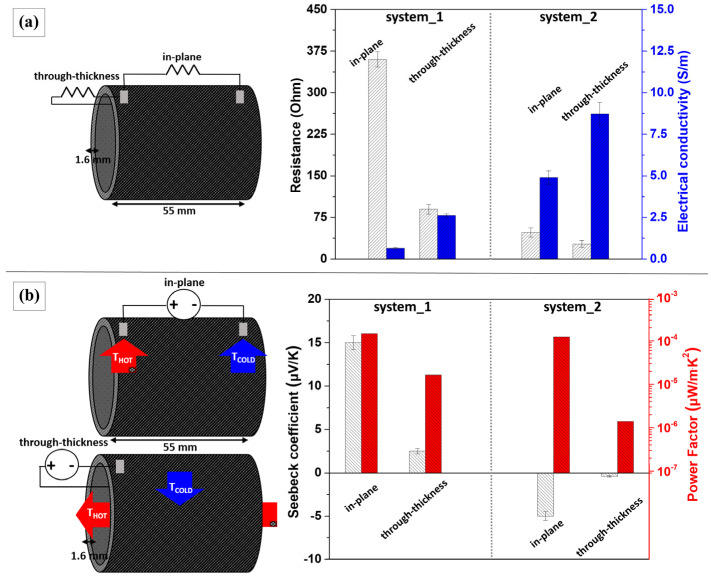
Measuring configurations and comparison graphs of the (**a**) electric and (**b**) thermoelectric response for in-plane and through-thickness measurements resulting from the two different systems of multifunctional GFRP tubes.

Figure 9 illustrates schematically the corresponding electric and thermoelectric measurement configurations for the tested multifunctional GFRP tubes with geometric characteristics of 55 mm length (L), 45 mm diameter (d) and 1.6 ± 0.2 mm thickness (t). In Figure 9a are presented both the in-plane (interelectrode distance of 55 mm) and through-thickness (interelectrode distance of 1.6 mm) two-probe electrical resistance (*R*) mean values at RT for system 1 and system 2. More specifically, for system 1 the in-plane electrical resistance was 360 ± 12.1 Ohm, while the through-thickness was 90 ± 5.7 Ohm. Correspondingly, for system 2 the in-plane *R* was 48 ± 7.3 Ohm and the through-thickness was 27 ± 4.4 Ohm. The exported electrical conductivity (*σ*) values were determined for each case by the customized formula: *σ* = L/*R*·π·(r_1_^2^ − r_2_^2^) [50], where r denotes the radius; for system 1 this was calculated to be 1.42 ± 0.06 S/m (in-plane) and 5.67 ± 0.24 S/m (through-thickness), whereas for system 2 it was calculated to be 10.63 ± 0.29 S/m (in-plane) and 18.91 ± 0.32 S/m (through-thickness). It is worth mentioning that the bulk through-thickness measurements presented the highest *σ* values for both systems. Notably, a 233% difference between system 1 and system 2 was observed. Comparatively, for all cases, the obtained values from system 2 were approximately one order of magnitude higher than those of system 1. The aforementioned difference in electrical values is believed to originate in the superior wetting properties on account of the chemically compatible polymeric dispersant [51] and resin impregnation for the coated GF-MWCNT:PVP tows (system 2). Subsequently, this fact could lead to more satisfactory compaction during the thermal curing polymerization process of the GFRP composite tubes. Additionally, experimental bulk thermal conductivity (κ) at RT for system 1 was found to be 0.63 ± 0.004 W/m·K, and for system 2, 0.75 ± 0.002 W/m·K, indicating a difference of 19%. The mean κ value of the reference conventional GFRP tube samples was 0.55 ± 0.006 W/m·K. Similar findings were revealed by Li and coworkers for amine-functionalized MWCNT electrophoretic deposited onto continuous reinforcing GFs within a cyanate ester epoxy matrix [52]. Therefore, the multifunctional GFRP tubes from system 2 appeared to be more electrically and thermally conductive, due to a higher volume fraction of modified reinforcement, which also implies more internal conductive paths between the highly conductive reinforcements. On the other hand, for system 1, a generally poor-quality wetting was observed, which is also evident from the comparatively greater thickness variance of the respective samples owing to the accumulation of a higher matrix percentage, rather than reinforcement in the final volume fraction for the tubes.

Additionally, Figure 9b presents the average experimental values of the Seebeck coefficient (*S*) and the corresponding calculated thermoelectric power factor (*PF* = *σ*·*S*^2^) for each case. Thermoelectric energy conversion based on the Seebeck effect permits the generation of electricity directly from a material upon its exposure to temperature differences. The Seebeck effect relies on the conversion of temperature gradient (Δ*Τ*) to electrical voltage (Δ*V*), and the Seebeck coefficient can be positive (p-type) or negative (n-type), depending on the type of the semi-conductor employed as active element [53]. Desired requirements for an overall high thermoelectric efficiency is the combination of high *S* and *σ* values, coupled with low κ characteristics at material level, and is classified by the dimensionless thermoelectric figure of merit ((*ZT* = (*σ*·*S*^2^)·T/κ)) [54]. The thermoelectric sensitivity (e.g., thermocouple voltage per unit temperature difference) for system 1 exhibited a positive Seebeck coefficient of +15 ± 0.44 μV/K for the in-plane direction with a *PF* of 3.19 × 10^−4^, and +2.5 ± 0.32 μV/K for the through-thickness direction with a *PF* of 3.55 × 10^−5^. Conversely, system 2 exhibited a negative Seebeck coefficient of −5 ± 0.35 μV/K for the in-plane direction with a *PF* of 2.66 × 10^−4^, and −0.4 ± 0.05 μV/K for the through-thickness direction with a *PF* of 3.03 × 10^−6^. It should be highlighted that the Seebeck coefficient consists of an inherent material physical property [55], arising only from the MWCNT type. The values’ fluctuation between the in-plane and through-thickness direction strongly depends on the geometrical parameter of the variant interelectrode distance, which impacts the real absolute Δ*T* within the volume of the composite structure, as also described in a past study [54]. Representatively, the derived *ZT* value of the bulk through-thickness for system 1 was 2.24 × 10^−8^, while for system 2 it was 1.60 × 10^−9^. As mentioned, GF-MWCNT:SDBS presented a p-type thermoelectric character, which is typically expected for alike carbon allotropes [56], on account of their tendency to strongly adsorb oxygen atoms owing to their hexagonally packed structure. On the other hand, GF-MWCNT:PVP showed an n-type thermoelectric behavior, indicating that the thermoelectric effect was governed by the diffusion of electron charge carriers. This fact could be attributed to an electron donation mechanism, causing a majority of electrons to arise from nitrogen heteroatoms in PVP side groups [57], which is able to shift the Fermi energy in relation to its initial energy state [58]. 

Indicatively, in a continuous fibrous conductive reinforcement within a polymer matrix composite structure, the in-plane direction is dominated by the fibers and the through-thickness direction is dominated by the polymer matrix. In spite of the GFRP attractive through-thickness electrical conductivity, both the Seebeck coefficient and thermal conductivity values are not efficient enough for thermoelectric applications, resulting in an inherent low thermoelectric power [5]. However, there has been limited investigation into tailoring the thermoelectric properties, which are important for electrical power generation, heating and cooling.

## 4. Conclusions

In the research work at hand, an industrial cross-scale manufacturing approach was implemented towards the production of multifunctional GFRP composite tubes. For this purpose, a continuous pilot filament winding line was designed, able to exploit a wet chemical deposition technique in order to modify microscale reinforcing GFs with MWCNT-based nanostructured coatings. Two kinds of aqueous-based MWCNT inks were developed with divergent dispersing agent and concentration, which were further utilized for the bath coating stage. The resulting GF-MWCNT hierarchical composite systems exhibited significant differences in their final mechanical, electrical, thermal and thermoelectric capabilities. More specifically, tube compression tests revealed a dissimilar ability to absorb energy at a constant rate through progressive deformation coupled with an alternative fracture resistance for the two compared systems. For lateral compression in particular, the MWCNT coatings comprising a polymeric dispersant minorly influenced the mechanical response of the produced tubes. The crashworthiness indicators of the multifunctional tubes displayed a slight 5% variation from the respective reference values, coupled with more ductile characteristics. The absorption capacity in the axial direction is remarkably greater than in the longitudinal direction. Absorbed energy is a crucial design criterion for many applications, such as the automotive sector. However, for the commercialization of a composite tube as a fluid transport pipe, the PCF is considered as the critical design parameter, as beyond that limit the structure loses its load-bearing capacity. It can be concluded that the experimental testing procedure showed a consistent repeatability and the obtained reliability index values are considered safe. This fact demonstrates that the production of multifunctional GFRP composite tubes with filament winding method appears to be a low-cost procedure with high productivity and scalability. Finally, the imparted bulk electrical, thermal and thermoelectric functionalities of the hierarchical GFRP tubes possess the potential to render them capable of thermal control, energy saving and hazard mitigation in large-volume applications. As an illustration, for the bulk electrical and thermal conductivity values, as well as the Seebeck coefficient factor, the corresponding tubes exhibited a deviation of 233% and 19% and an opposite semi-conducting sign denoting a p- and n-type character, respectively. Multifunctional GFRP tubes could take advantage of their newly introduced superior bulk electrical conductivity to boost safety factors by reducing spark risk or to become heatable for humidity and/or ice removal. The proven highly scalable methodology and further optimization strategies of the cross-scale reinforced multifunctional GFRP tubes could open new routes for monolithic composite structures or components with tailored mechanical performance, anti-static properties, accumulated thermal energy dissipation and energy-harvesting attributes.

## Figures and Tables

**Table 1:** Crashworthiness indicators under lateral and axial crushing.

Tubes	Lateral Crushing		Axial Crushing	
	PCF (kN)	EA (J)	PCF (kN)	EA (J)
reference	3.01 ± 0.06	62.52 ± 2.05	30.52 ± 1.74	551.50 ± 92.58
system 1	1.53 ± 0.26	51.56 ± 5.63	15.44 ± 2.64	389.93 ± 73.78
system 2	2.82 ± 0.16	60.34 ± 1.39	20.67 ± 2.82	399.79 ± 66.77

## Data Availability

The original contributions presented in the study are included in the article/Supplementary Material, further inquiries can be directed to the corresponding author.

## Supplementary Materials

The following supporting information can be downloaded at: https://www.mdpi.com/article/10.3390/polym16121754/s1, Figure S1: The examined coupons of the three different GFRP tubular systems with their special geometrical features; Table S1: Representative dimensional values for the tested tubular systems; Figure S2: Representative graph defining the crashworthiness criteria; Figure S3: Typical load-displacement curves of tubes under crushing noting the three distinct regions and the respective transition points for (a) lateral and (b) axial loading; Table S2: Crashworthiness indicators for the tube systems upon lateral crushing; Table S3: Crashworthiness indicators for the tube systems upon axial crushing.
