# Supplementary material for: Cross-Scale Industrial Manufacturing of Multifunctional Glass Fiber/Epoxy Composite Tubes via a Purposely Modified Filament Winding Production Line
Source: Polymers (Basel). 2024 Jun 20;16(12):1754. doi: 10.3390/polym16121754 (PMC11207220; doi:10.3390/polym16121754)
Supplement: Supplementary file 1 [file polymers-16-01754-s001.zip › polymers-3036106-supplementary.pdf]

Supporting Information

Cross-scale industrial manufacturing of multifunctional glass fiber/epoxy composite tubes via a purposely modified filament winding production line

George Karalis [1], Lampros Koutsotolis [1], Angelos Voudouris Itksaras [1], Thomai Tiriakidi [2], Nikolaos Tiriakidis [2], Kosmas Tiriakidis [2], Alkiviadis S. Paipetis [1,*]

1 Department of Materials Science and Engineering, University of Ioannina, 45110 Ioannina, Greece; gkaralis@uoi.gr; l.koutsotolis@uoi.gr; a.voudouris@uoi.gr; paipetis@uoi.gr

2 BT Composites S.A., 53100 Florina, Greece; thomai@btcomposites.gr

* Correspondence: paipetis@uoi.gr; Tel.: (+30)-265100-8001

The coupons extracted from longer tubes had an inner diameter of 45 mm, as determined from the filament winding rotating mandrel and were cut into specimens 55 mm long. Geometrical parameters of the specimen also include the thickness (t), diameter (D) and mass (m). The tube diameter is defined as the mean value of the outer and inner diameters, *i.e.*: $D=D_{inner}+t/2$. It should be noted here that the diameter to the tube cell wall thickness (D/t) is an important design parameter in composite tubular structural components, as it affects their failure behavior [1,2]. The geometrical characteristics of the three different tube systems are shown in Figure S1 and summarized in Table S1. The recorded values are the average of 10 measured tubes belonging to each specimen system. The coated GFs brought upon a minor change in the weight of the corresponding tubes. More specifically, system 1 was 3.85% heavier and 4.31% thicker than the reference tubes, whereas for system 2 both weight and thickness were reduced by 2.77% and 4.63%, respectively. This is in good agreement with the respective fiber volume fractions. Fiber volume ratio of system 1 was 5% less than the reference, while of system 2 was 1.5% more, leading respectively to thicker and thinner tube. The fiber volume ratio was calculated using the formula $V_f = v_f/v_c = (m_f/\varrho_f)/(m_f/\varrho_f + m_m/\varrho_m)$, where V_f is the fiber volume fraction, v_f is the volume of the fibers and v_c is the volume of the composite. To derive the volumes the measured weight of the GFs and the matrix ($m_m=m_c-m_f$) was used (m_f), according to their respective densities. The density of the E-GF roving was taken by the manufacturer's technical datasheet. The density of the cured epoxy resin system was calculated by manufacturing a small rectangular specimen and measuring its mass and dimensions. The fiber volume fractions were calculated equal to 66.21%, 62.85%, and 67.21% for the reference, system 1 and system 2, respectively. It is noted that the MWCNT coating of the modified GF tows was almost completely removed after the acid bath and the water washing. Therefore, only minorly affecting the corresponding fiber volume fractions. The smaller volume fraction of the system 1 tubes with respect to the reference ones may slightly decrease their mechanical performance. However, their significant mechanical degradation owns to the MWCNT-based ink used, that alters the failure mechanisms of the tubes.

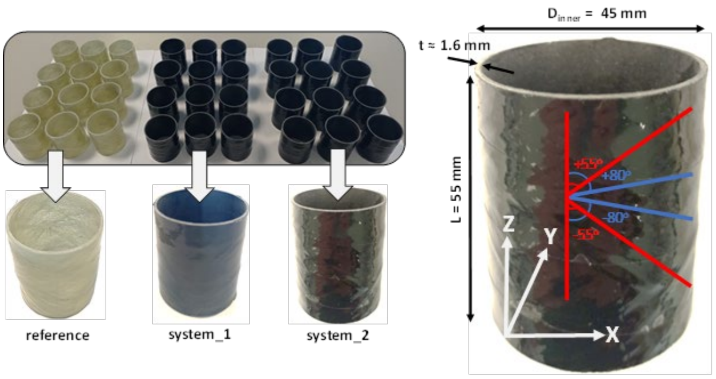

Figure S1. The examined coupons of the three different GFRP tubular systems with their special geometrical features.

Table S1. Representative dimensional values for the tested tubular systems.

Tubes	L (mm)	m (g)	t (mm)	D_{inner} (mm)	D_{outer} (mm)	D (mm)	D/t
reference	55.00	23.20 ± 0.17	1.60 ± 0.03	45.00	46.60 ± 0.03	45.80 ± 0.02	28.57 ± 0.58
system 1	55.00	24.09 ± 0.31	1.67 ± 0.05	45.00	46.67 ± 0.05	45.83 ± 0.02	27.49 ± 0.79
system 2	55.00	22.56 ± 0.21	1.53 ± 0.04	45.00	46.53 ± 0.04	45.76 ± 0.02	30.02 ± 0.76

Regarding the hydrothermal durability investigations, saturation occurs when the moisture content changes by less than 0.020% in two consecutive measurements. Moisture absorption is calculated by the relative weight gain of the specimens. $W=(m_i-m_0)/m_0 \cdot 100\%$, where W is the moisture content (%), m_0 is the baseline specimen mass (g) *i.e.*, the mass of the specimen after drying and m_i the current specimen mass. The weight of the samples was measured on a Shimadzu libror AEU-21 enclosed analytical balance with four significant digits. Prior to hydrothermal aging the samples were vacuum dried at 50°C until zero moisture content. The two edges of the tubes exposed to aging were sealed using silicone so that moisture absorption occurs only from the circular surfaces. Consequently, moisture absorption can be considered be one-dimensional, following Fick's second law of diffusion: $\partial W/\partial t=D_z \cdot (\partial^2 \cdot W)/\partial z^2$, where W is the moisture concentration ($g \cdot m^{-3}$), t is the time (s) and z is the direction (m) at which the moisture diffusion occurs, in this case the thickness of the tube. D_z is the diffusion coefficient ($m^2 \cdot s^{-1}$), which is a material property and constant through the thickness of the material.

For the compression tests, the specimens were placed in the center of two flat parallel platens to be crushed. The top platen moved downwards, while the bottom one remained fixed. In the case of axial compression, the upper and lower ends of the specimens were grinded to become even and completely parallel to each other and thus perpendicular to the loading direction to avoid possible eccentricities during testing. The maximum displacement was set at 40 mm corresponding to a strain of 72.72% and 87.32% for axial and lateral compression respectively. This displacement almost coincides to the displacement at which the material enters the densification stage. A constant maximum displacement for all tested samples is deemed necessary, as it is a parameter that affects the crashworthiness indicators.

The crushing behavior of composite tubes can be described and quantified with the assistance of several indicators called crashworthiness criteria or characteristics or indicators. The deriving of the indicators is summarized in Figure S2 [3]. Peak Crushing Force (PCF) is the maximum force that the material can bear before the first failure occurs. Energy absorption (EA) is the total strain energy absorbed by the material during the crushing procedure. EA, being the area under the load-displacement curve can be directly calculated by integration. $EA = \int_0^d F(x)\,dx$, where F(x) is the load as a function of the displacement and dx is the total displacement. Specific Energy Absorption (SEA) is defined as the ratio between the SEA and the mass and it used to quantify the mass efficiency, $SEA = \frac{EA}{m}$. Mean Crushing Force (MCF) is defined as the ratio between the SEA and the total crushing displacement and it used to measure the loading capacity efficiency. In the load-displacement curve MCF is the mean value around, which the force oscillates during the plastic collapse stage, $MCF = F_{mean} = \frac{EA}{m}$. Crushing Force Efficiency (CFE) quantifies the uniformity of the crushing load, $CFE = \frac{MCF}{PCF}$ [3].

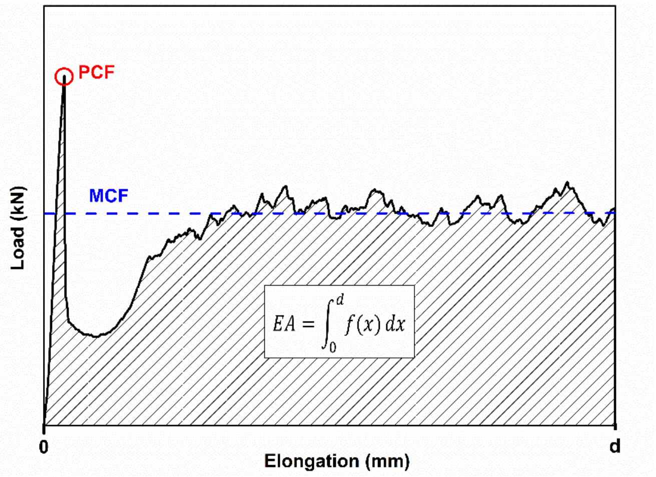

Figure S2. Representative graph defining the crashworthiness criteria.

As is depicted in Figure S3 the load-displacement curves under both axial and lateral crushing follow a characteristic pattern that can be divided into three distinct regions. These regions correspond to different deformation and energy absorption mechanisms. More precisely, initially the material behaves linearly elastically (Region I), followed by a progressive failure process (Region II) and finally the densification or self-contraction of the material occurs (Region III). The initial elastic region corresponds to the global linear elastic deformation of the tube and is controlled by the bending of its walls. The elastic region is characterized by reversible deformation and high modulus of elasticity. The tube can bear the compressive load until the PCF is reached. At this point the first failure occurs and the load drops sharply to lower values. PCF is the transition point to Region II, called progressive crushing or progressive collapse stage. In this region, although the deformation gradually increases, the load bearing capacity exhibits a rather stable trend and oscillates around the value of the mean crushing force. This oscillating plateau region corresponds to the progressive collapse of its walls occurring due to the propagation of existing cracks and delamination, as well as the formation of new ones. This process continuously builds up and releases strain energy. Finally, from a deformation onwards, load starts to increase again in an almost exponential manner. This occurs when the failure process reaches its saturation and the material experiences densification. This behavior approaches the linear elastic behavior of the bulk material.

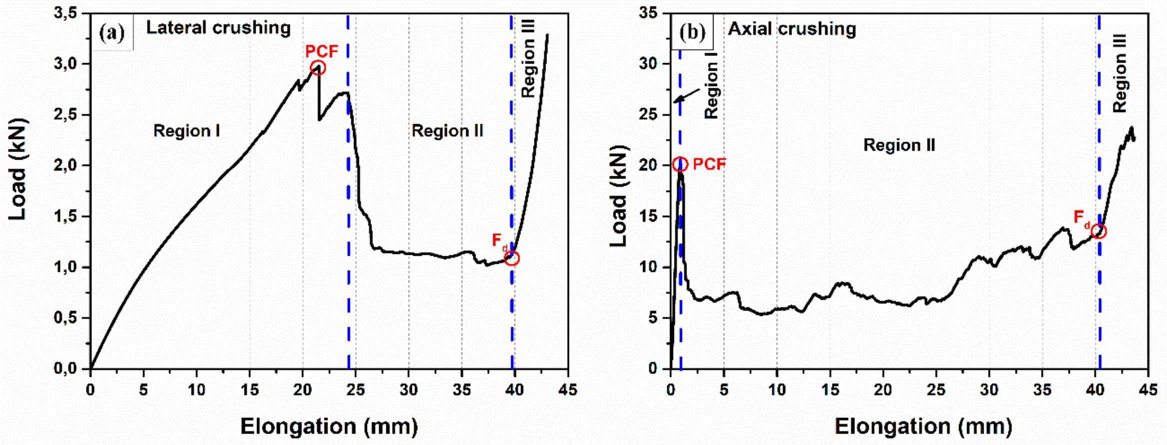

Figure S3. Typical load-displacement curves of tubes under crushing noting the three distinct regions and the respective transition points for (a) lateral and (b) axial loading.

Although the load-displacement curves follow the same qualitative pattern for both loading directions, there are significant quantitative differences between them. In lateral crushing (Figure S3a, Table S2) the elastic region is the prevailing part of the curve. Initially the tube exhibits Hookean behavior, but as the loading progresses further it deviates from linearity although it still remains elastic. During the last stage of the elastic region, just before the PCF is developed, small scale damage in the form of delamination appears as denoted by the small drops of the load in the curve. The large elastic

deformation obtained during lateral crushing is a direct consequence of the geometry, namely the bending of its cylindrical walls. The sharp drop of load after the PCF is due to the failure of the specimen by the formation of two fracture lines located at 90° angle with the flat plates of the testing machine. Normally two load drops can be observed, one at the PCF and one at a slightly lower force occurring immediately after. Those two drops are attributed to the non-simultaneous development of a pair of plastic hinges. From this point on the tube ceases to be a single cylindrical component, but consists of two almost independent semi-cylindrical sections. Consequently, the plateau region corresponds to the bending of those sections. Delamination initiates from the fracture lines and propagates throughout the specimen. At the contact points of the tube with the testing machine platens high strain is present. Despite this fact only individual GF filaments get fractured, that do not lead to the formation of plastic hinges there. Apart from delamination propagation no other damage mode occurs during the plateau region, hence the stability of the load. Finally, the load increases again when the two sections of the tube flatten and come into contact with each other during the densification stage [1]. In axial crushing (Figure S3b, Table S3) the elastic part of the curve is small and limited only to the early deformation stages. This region is characterized by high stiffness, as the walls of the whole tube bear the compressive load. The response of the tube is initially linear elastic, but a deviation from linearity can be observed, as the mechanical testing progresses. When the peak load value is reached, instability and extensive damage occur in the weakest cross-section of the tube and a failure zone is formed. Thus, the load bearing capacity drops sharply to lower levels. From this point onwards the specimen experiences progressive collapse. This region is much more pronounced in axial crushing and constitutes the dominant part of the load-displacement curve. In this region the loading capacity has a relative stable trend and exhibits small-scale fluctuations around the MCF. Drops in load represent local plastic failures, which release the stored strain energy. Then, the remaining healthy part of the tube bears the compressive load and strain energy builds up again. The consecutive load fluctuations correspond to the crushing of the next successive area around the first failure zone. The fact that the collapse stage dominates the load-displacement curve, as well as the relative stability of the load at this of this stage imply that the deformation and damage growth occur in a stable and progressive manner. As a result, most of the strain energy is absorbed during this stage, happening indeed at a constant controllable rate [2]. When the whole tube has plastically collapsed the densification stage takes place.

Table S2. Crashworthiness indicators for the tube systems upon lateral crushing.

Tubes	PCF (kN)	EA (J)	SEA (J/g)	MCF (kN)	CFE
reference	3.01 ± 0.06	62.52 ± 2.05	2.67 ± 0.07	1.56 ± 0.05	0.52 ± 0.02
system 1	1.53 ± 0.26	51.56 ± 5.63	2.13 ± 0.20	1.29 ± 0.14	0.85 ± 0.07
system 2	2.82 ± 0.16	60.34 ± 1.39	2.68 ± 0.05	1.51 ± 0.03	0.54 ± 0.04

Table S3. Crashworthiness indicators for the tube systems upon axial crushing.

Tubes	PCF (kN)	EA (J)	SEA (J/g)	MCF (kN)	CFE
reference	30.52 ± 1.74	551.50 ± 92.58	23.93 ± 3.94	13.79 ± 2.31	0.45 ± 0.08
system 1	15.44 ± 2.64	389.93 ± 73.78	16.37 ± 3.09	9.75 ± 1.84	0.63 ± 0.05
system 2	20.67 ± 2.82	399.79 ± 66.77	17.75 ± 3.15	9.99 ± 1.67	0.50 ± 0.13

References

1. Gupta, N.K.; Abbas, H. Lateral collapse of composite cylindrical tubes between flat platens. *Int. J. Impact Eng.* **2000**, *24*, 329–346, doi:10.1016/S0734-743X(99)00173-6.
2. Li, S.; Guo, X.; Li, Q.; Ruan, D.; Sun, G. On lateral compression of circular aluminum, CFRP and GFRP tubes. *Compos. Struct.* **2020**, *232*, 111534, doi:10.1016/j.compstruct.2019.111534.
3. Cui, Z.; Liu, Q.; Sun, Y.; Li, Q. On crushing responses of filament winding CFRP/aluminum and GFRP/CFRP/aluminum hybrid structures. *Compos. Part B Eng.* **2020**, *200*, 108341, doi:10.1016/j.compositesb.2020.108341.
